# A Low-Cost Passive Acoustic Toolkit for Underwater Recordings

**DOI:** 10.3390/s25237306

**Published:** 2025-12-01

**Authors:** Vassilis Galanos, Vasilis Trygonis, Antonios D. Mazaris, Stelios Katsanevakis

**Affiliations:** 1 Department of Marine Sciences, School of the Environment, University of the Aegean, University Hill, 81100 Mytilene, Greece; 2 Department of Ecology, School of Biology, Aristotle University of Thessaloniki, 54124 Thessaloniki, Greece

**Keywords:** passive acoustic equipment, piezoelectric sensor, underwater recording, low-cost hydrophone, scientific outreach

## Abstract

Passive acoustic monitoring is a key tool for studying underwater soundscapes and assessing anthropogenic impacts, yet the high cost of hydrophones limits large-scale deployment and citizen science participation. We present the design, construction, and field evaluation of a low-cost hydrophone unit integrated into an acoustic toolkit. The hydrophone, built from off-the-shelf components at a cost of ~20 €, was paired with a commercially available handheld recorder, resulting in a complete system priced at ~50 €. Four field experiments in Greek coastal waters validated hydrophone performance across a marine-protected area, commercial port, aquaculture site, and coastal reef. Recordings were compared with those from a calibrated scientific hydrophone (SNAP, Loggerhead Instruments). Results showed that the low-cost hydrophones were mechanically robust and consistently detected most anthropogenic sounds also identified by the reference instrument, though their performance was poor at low frequencies (<200 Hz) and susceptible to mid-frequency (3 kHz) resonance issues. Despite these constraints, the toolkit demonstrates potential for large-scale, low-budget passive acoustic monitoring and outreach applications, offering a scalable solution for citizen scientists, educational programs, and research groups with limited resources.

## 1. Introduction

Sound is a crucial component of the marine environment [1,2] and can be used to study animal life in the ocean [3,4]. Many marine animals have evolved ways to use sound production and hearing in behavioral facets that are critical to their survival, such as cetaceans, marine invertebrates and numerous fish [3,5,6]. Yet, in the oceans, anthropogenic low-frequency noise (<1 kHz) from shipping has steadily increased over the last 50 years [7,8,9] at a concerning average rate of 3 dB per decade [10]. Indeed, most human activities in the marine environment produce sound, and this pervasive input is increasingly altering the marine acoustic environment [11]. Depending on temporal patterns of traffic density, proximity to shipping routes, and acoustic propagation conditions [12], anthropogenic noise can have detrimental effects on marine biota and may disrupt entire biocommunities [13,14,15,16,17].

In the European Union (EU), low levels of underwater noise are a criterion for achieving ‘good environmental status’, as defined by the Marine Strategy Framework Directive (MSFD) in Descriptor 11: ‘Energy, including underwater noise’ [18]. Furthermore, each member state of the EU is required to research, monitor and report on the levels and spatiotemporal distribution of impulsive and continuous underwater noise [19]. In the open waters of the Mediterranean, the main sources of anthropogenic noise are the low-frequency sounds radiated from commercial ships and fishing vessels [20], while coastal waters are also subjected to small boat noise from recreational activities during the summer months [21,22]. Coastal marine-protected areas (MPAs) implement various marine traffic restrictions and no-fishing policies [23,24], but their proximity, and often overlap, with tourist hotspots, coupled with the pervasive nature of underwater noise, limits their efficiency in safeguarding against this stressor [25,26,27].

The characterization of marine soundscapes and the study of underwater sound are, therefore, key for management bodies to develop informed noise mitigation policies [28,29,30]. To this end, passive acoustic monitoring (PAM) methods are particularly suited, either using fixed acoustic stations [31,32,33], moored oceanographic buoys [34], free-drifting calibrated hydrophones [35] or ad hoc spot measurements [36]. Even though these methods can provide valuable and high-quality measurements, their data collection efforts are often limited by financial and personnel availability constraints when aiming for large-scale coverage. In an effort to pursue low-cost alternatives to expensive underwater recording equipment, various initiatives have developed custom versions of low-cost hydrophones, available either commercially with underwater housing, supporting software and duty-cycle options [37] or as home-built do-it-yourself (DIY) designs based on off-the-shelf piezoelectric (PZ) elements for on-site recordings [38,39,40,41].

The aim of this study was to develop and test, in-depth, under different usage scenarios, an easy-to-construct, low-cost hydrophone intended for large-scale acoustic sampling by citizen scientists and researchers alike. Its performance for demanding research applications such as marine traffic measurements and long-term soundscape monitoring was assessed across four field experiments at coastal sites, designed to capture both anthropogenic and biological sounds. All field recordings of the low-cost unit were compared with a scientific-grade hydrophone (the SNAP autonomous recorder from Loggerhead Instruments), while performance tests were also conducted in the laboratory using a Lubell underwater loudspeaker for playback of artificial signals and delphinid sounds.

## 2. Materials and Methods

### 2.1. Hardware Components and Hydrophone Construction

The proposed setup is part of a low-cost acoustic toolkit comprising a hydrophone and a commercially available handheld recorder. The recorder can optionally be replaced by a mobile phone for easy-to-conduct field recordings. This hydrophone offers a cost-effective design (approximately 20 € per unit, plus 30 € for the recorder) and a simple assembly that enables straightforward acoustic sampling.

**Figure 1:**
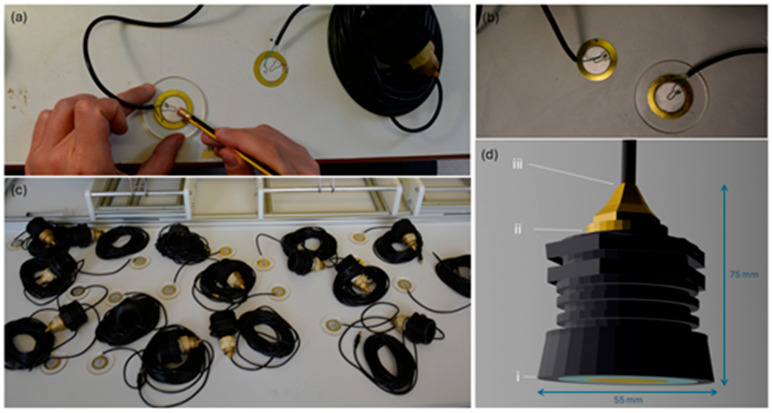
Construction steps of the low-cost hydrophones developed in this study: (**a**) Coupling of the piezoelectric element onto the plexiglass disk using cyanoacrylate glue. (**b**) The resulting acoustic element after the gluing process. (**c**) A hydrophone production batch before water sealing. (**d**) Three-dimensional model of the low-cost hydrophone, rendered in the open-source Blender software (v. 4.5.2 LTS); the annotations (i–iii) mark points where water sealants were applied.

Central to this low-cost hydrophone design is a thin piezoelectric disk 35 mm in diameter (BeStar: FT-35T-2.6A1, for more technical information, see Appendix A), chosen for its low cost and availability in the market, identical to the PZ elements commonly used in gift cards, acoustic guitar pickups, and electronic buzzers. After soldering it to an off-the-shelf audio cable, the PZ disk is glued with cyanoacrylate (‘super’) glue onto a plexiglass disk of 4 mm thickness. The latter is then superglued to a PVC threaded nipple and acts both as the face of the hydrophone and as a lid for the enclosure (Figure 1a–c). At the back (recorder-facing) side of the hydrophone, the audio cable exits the enclosure through a brass pipe fitting that is screwed into the PVC thread and closely matches the cable’s diameter at its narrowest end, ensuring a robust tail-end which strengthens the enclosure. Finally, all connection points of the hydrophone are sealed to prevent water influx using sealant materials that are commonly used in marine applications. Specifically, the PVC-to-plexiglass connection (Figure 1d-i) is sealed with a thick layer of Sikaflex-291i adhesive sealant, taking care not to cover the hydrophone’s face (outer side of the plexiglass disk) and the PZ element that sits behind it. The threaded part (Figure 1d-ii) is sealed with both Teflon tape and liquid Teflon, and the exit point of the audio cable (Figure 1d-iii) is protected with a layer of adhesive sealant and heat shrink tubing. The resulting unit offers adequate mechanical strength and enough weight to achieve slightly negative buoyancy. A full list of assembly components with indicative prices is provided in Appendix A (Table A1).

### 2.2. Laboratory Recordings

To assess the hydrophone’s sensitivity across different frequencies, the custom-made device was placed in an open-top seawater tank (113 × 40 × 63 cm) together with a Lubell LL916C underwater loudspeaker used for signal playback. The two devices were positioned at opposite ends of the tank, 80 cm apart, with the hydrophone’s sensing surface oriented orthogonally to the loudspeaker’s emission axis. The Lubell loudspeaker (frequency response 0.2–23 kHz) was powered by a Peavey Electronics IPA 300T amplifier (frequency response 0.04–20 kHz) connected to the audio output of a laptop computer.

An artificial sine-wave signal was created with the tone generator tool of the Audacity software (v3.7.5) that spanned the frequency range of 200 Hz to 20 kHz. Each tone had an amplitude of 0.9 (on a 0–1 scale) and a duration of 2 s, with frequency increments of 50 Hz for the frequency range 200–1000 Hz, 100 Hz for the 1100–4000 Hz range, and 250 Hz for the 4250–20,000 Hz range. These tones were saved as a continuous 16-bit uncompressed audio (WAV) file that was subsequently transmitted underwater (followed by 5 s of silence) while being recorded by the custom-made hydrophone via a Tascam DR-05X handheld recorder. For each tone recorded, the root-mean-square (RMS, 0.2–20 kHz) amplitude was calculated from the central 1 s segment of the recorded signal, and received levels were expressed in dB relative to the RMS of 1 s of ‘silence’. The output of this recording session was then compared against the sound pressure level (SPL) curve of the Lubell LL916C, digitized from the manufacturer’s specifications. Using the same experimental setup, additional tests were conducted with playback of bottlenose dolphin (*Tursiops truncatus*) whistles previously recorded in the wild [42].

### 2.3. Experimental Field Recordings

**Figure 2:**
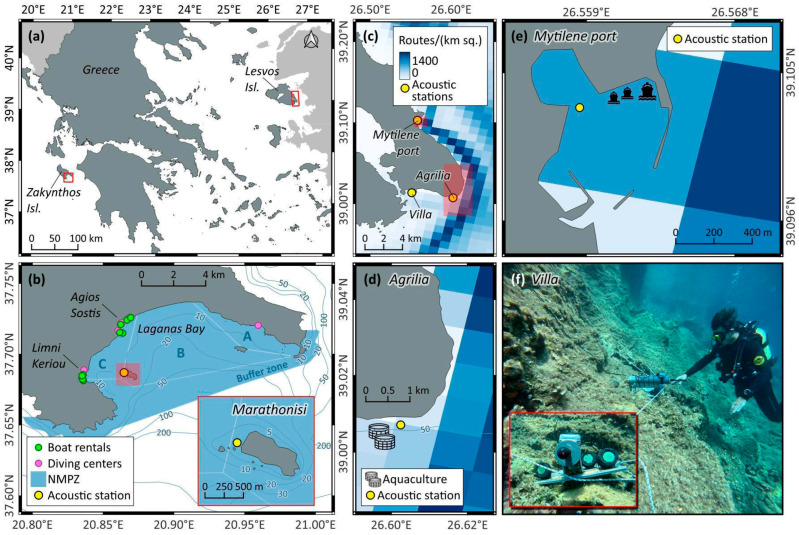
(**a**) Map of Greece, showing the Zakynthos and Lesvos islands where experimental recordings took place; (**b**) The Marathonisi recording site inside the National Marine Park of Zakynthos; (**c**–**e**) The recording sites of Agrilia, Mytilene port, and Villa at Lesvos Island; (**f**) Underwater images of the acoustic station deployed at Villa; an extra (third) custom-made hydrophone visible in the inset image was for redundancy and not used herein.

Two custom-made uncalibrated hydrophones were experimentally tested at four coastal sites of the Aegean and Ionian Seas (Greece): Marathonisi Islet at the National Marine Park of Zakynthos (NMPZ), the port of Mytilene at Lesvos Island, and the sites of Agrilia and Villa at Southeast Lesvos (Table 1, Figure 2).

During these test recordings, one custom-made hydrophone (hereafter referred to as ‘Nemo-1′) was always coupled with a Philips DVT 1120 handheld recorder that is part of the acoustic toolkit. The recorder of the second custom-made hydrophone (‘Nemo-2′) varied by site and was either a Tascam DR-05X or an M-Audio MicroTrack II. The SNAP autonomous underwater recorder from Loggerhead Instruments, equipped with the HTI-96-Min acoustic sensor (sensitivity −170 dB re 1V/μPa), was used in all sessions as a reference, factory-calibrated scientific hydrophone. For ease of deployment, all acoustic devices were mounted on the same custom-built stainless-steel base, which weighed approximately 8 kg in air (Figure 2f). A summary of recording settings per test session is provided in Table 1, while the recording sites are described in the sections that follow.

#### 2.3.1. Recording Site 1: Marathonisi Islet at NMPZ

The National Marine Park of Zakynthos covers 83.3 km^2^ of marine-protected area along the south shores of Zakynthos Island (Ionian Sea, Greece). It fully encompasses Laganas Bay, a shallow water (<50 m) embayment with soft substrate dominated by unvegetated sandy beds and *Posidonia oceanica* meadows [43]. This MPA hosts one of the most important rookeries of the loggerhead sea turtle *Caretta caretta* in the Mediterranean [44,45], but is also subjected to severe anthropogenic pressures due to the recreational and economic importance of the area. The sandy beaches that stretch along inner Laganas Bay are heavily visited during summer, while nearshore infrastructure and coastal sites of high aesthetic value support a thriving tourism industry that peaks from June to August [46]. Leisure boat traffic is substantial during this season, and includes rigid inflatables, speedboat rentals, and hard-hulled eco-tourism boats that mostly launch from the ports of Limni Keriou and Agios Sostis (Figure 2b).

The zoning scheme of NMPZ keeps most speedboats away from the key nesting beaches of *C. caretta* at East Laganas Bay, but channels them near Marathonisi Islet and the rocky shores of Southwest Zakynthos. To investigate the performance of our custom-made hydrophones in monitoring speedboat traffic in high-use coastal waters, a test recording session was conducted near Marathonisi Islet on 26 June 2025 (Figure 2b, inset). Using an anchored small boat, the acoustic station was positioned on the seafloor at 3 m depth and recorded from 11:20 to 14:20 local time (Table 1). Concurrent visual observations were also conducted by the researcher on site, including empirical boat distance estimates between the acoustic station and the speedboat at their nearest point, boat direction, and perceived speed. Leisure marine traffic was dense throughout the recording period, and the distance between the acoustic station and passing speedboats ranged from a few meters to over 300 m.

#### 2.3.2. Recording Site 2: Agrilia

A boat-based recording was carried out on 8 August 2025 in the vicinity of an aquaculture facility at Agrilia, Southeast Lesvos, in an effort to record the sounds produced by bottlenose dolphins (*Tursiops truncatus*) that frequent these waters [42]. The survey boat was anchored 200 m east of the aquaculture net pens (Figure 2d), and the acoustic station was lowered off the stationary boat to a depth of 7 m (Table 1). The recording started at 07:20 local time and was stopped after one hour due to worsening weather conditions. Although dolphins were neither sighted nor recorded, this fieldwork session captured the passage of a 195 m long ferry boat, northbound to Mytilene port. According to automatic identification system (AIS) data, which were monitored during recording (www.marinetraffic.com), the distance between the ferry and the acoustic station was approximately 2 km at its nearest point to the research boat.

#### 2.3.3. Recording Site 3: Mytilene Port

A test recording was conducted at the port of Mytilene (Figure 2e) on 10 June 2025, focused on inboard vessels. The acoustic station was deployed off the pier at 4 m depth (resting on the seafloor) and recorded the scheduled departure of a passenger boat (40 m in length) that travels daily between Mytilene and Ayvalik (Turkey). Recording settings for this session are listed in Table 1.

#### 2.3.4. Recording Site 4: Villa

The coastal underwater soundscape of the Villa site (Figure 2c) was recorded for 34.3 consecutive hours between 14 and 15 August 2025, starting at 13:10 local time. As reported by the local diving center that frequently visits this site, the rocky reefs and *Posidonia oceanica* patches of Villa host several soniferous fish, such as the brown meager, *Sciaena umbra* [47], the dusky grouper, *Epinephelus marginatus* [48], and various bottom-dwelling Scorpaenidae [49]. The acoustic station was fixed to the rocky substrate at a depth of 6 m (Figure 2f), while the handheld recorders were placed in a weatherproof case secured outside the seawater. Recording settings for this session are provided in Table 1.

### 2.4. Acoustic Data Processing

All acoustic recorders used in this study stored data in 16-bit WAV files, albeit with different file naming schemes and file storage lengths. For each test site, the initial preprocessing step consisted of manually synchronizing the concurrent recordings across all hydrophones, using the Raven Pro v1.6.5 sound analysis software [50]. The waveforms were visualized as separate channels in Raven Pro and were manually aligned in time using distinct audio marks (waveform peaks) that were added to the recording by the researcher on site. These marks were created by repeatedly hitting the steel station base with a metallic object in a rhythmic manner (both before deployment and after retrieval). The acoustic data files were cropped properly and renamed according to the SNAP file naming scheme (yyyymmdd_hhmmss), resulting in three audio datasets per recording site of identical timestamps and total duration. No filtering, resampling, or gain adjustments were applied to the raw data.

The synchronized acoustic series were then transformed by a fast Fourier transform (FFT) in Raven Pro, and the resulting spectrograms (N_FFT_ = 1024 samples, Hamming window, 50% overlap) were visually and aurally inspected to obtain an understanding of the dataset contents. Sounds of interest (e.g., fish sounds and passenger vessels) were manually annotated, and audio segments needing further processing were accordingly marked. For the Marathonisi recording, a detailed analysis was performed to manually mark all unique speedboat instances identified in the spectrograms, informed by the on-site visual observation log that recorded their vessel type and passage timestamp. Notwithstanding overlaps due to dense traffic, for each speedboat instance identified on the spectrogram’s time-frequency grid, a rectangular selection box was drawn that encompassed the sound of interest. The lower and upper frequency limits of the selection box were fixed at 30 Hz and 8 kHz, respectively, whereas the start and end time of the box defined the respective limits on the time axis. The following parameters were then extracted for each selection and examined with non-parameric Mann–Whitney tests for statistical differences between hydrophones: 90% duration, *dt*_90%_ = *t*_95%_ − *t*_5%_ (s), where *t*_5%_ and *t*_95%_ are the points in time that define the first and last 5% of the sound’s energy, respectively; peak frequency, *f*_peak_ (Hz); center frequency, *f*_50%_ (Hz), defined as the frequency that splits the annotation box into two parts of equal energy; 90% bandwidth, *Bw*_90%_ = *f*_95%_ − *f*_5%_ (Hz), and interquartile bandwidth, *Bw*_50%_ = *f*_75%_ − *f*_25%_ (Hz), where *f*_5%_, *f*_25%_, *f*_75%_, and *f*_95%_ are the frequencies that contribute to the first 5%, 25%, 75% and 95% of the sound’s energy, respectively. Subsequently, all speedboat selections were exported as separate WAVs, and the PAMGuide software [51] was used to compute their power spectral density (PSD).

To visually summarize the important contributors to the underwater soundscape over the 34.3 h recording at Villa, the PAMGuide software and custom MATLAB R2024a scripts were used to produce long-term spectral average (LTSA) plots with a 30 s averaging window. Root-mean-square (RMS) noise levels at 1/3-octave bands (TOLs) were also computed in PAMGuide for the Mytilene port and Agrilia sites. All spectrograms, PSD computations, TOLs, and LTSAs were produced in relative dB units (uncalibrated) for the custom-made Nemo hydrophones, while calibration data for the SNAP device were: 2 dB gain, clip level (peak-to-peak) 1.58 V, and hydrophone sensitivity −170 dB re 1V/μPa.

## 3. Results

### 3.1. Water Tank Recordings of Artificial and Biological Signals

**Figure 3:**
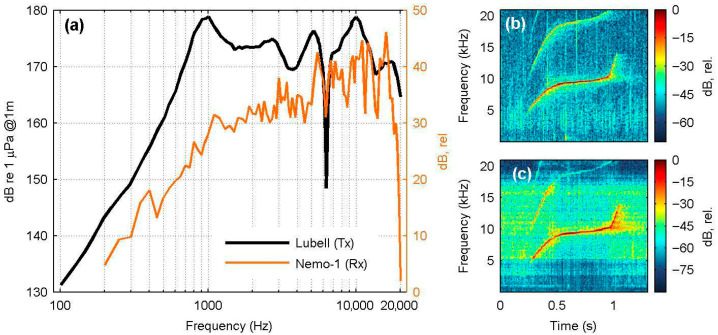
(**a**) Nominal plot of transmitted (Tx) sound pressure level vs. frequency of the Lubell LL916C loudspeaker (digitized from the manufacturer’s drawing, black line) and sound levels received (Rx) by the Nemo-1 hydrophone during the playback of the 0.2–20 kHz artificial signal. (**b**,**c**) Uncalibrated spectrograms of a transmitted *T. truncatus* whistle (**b**) and corresponding signal recorded by the Nemo-1 hydrophone (**c**).

Notwithstanding distortions of recorded signals due to boundary effects within the tank environment [52], the playback experiments showed that the received sound pressure levels at the Nemo-1 hydrophone were largely consistent with the theoretical output of the loudspeaker (Figure 3a). However, resonant peaks were observed at the 0.4 and 3 kHz regions, and the response of Nemo-1 was frequency-dependent, exhibiting several peaks and troughs at mid to high frequencies. Dolphin whistles were clearly captured by Nemo-1 (Figure 3b,c), demonstrating the potential of this device for low-cost cetacean acoustic monitoring.

### 3.2. Recordings of Recreational Boat Traffic at NMPZ

**Figure 4:**
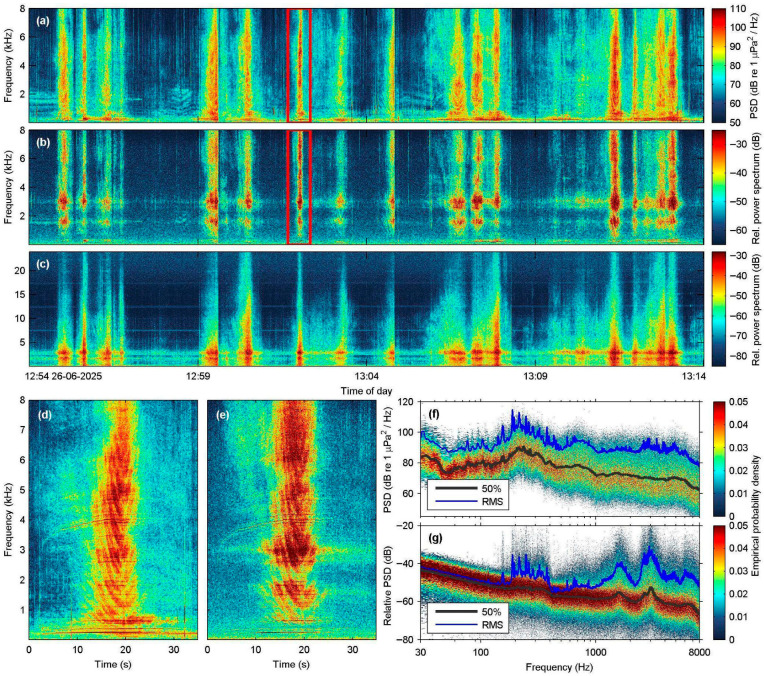
Spectral characteristics of speedboat traffic in the vicinity of the acoustic station at Marathonisi (NMPZ), as recorded with the SNAP and the Nemo custom-made hydrophones (12:54–13:14 local time); the red boxes indicate the speedboat instance shown in panels (**d**,**e**): (**a**) 20 min-long power spectral density (PSD) spectrogram produced from the SNAP data (N_FFT_ = 4096 samples, Hamming window, 50% overlap); (**b**,**c**) As in (**a**), but recorded with the Nemo-1 (**b**) and Nemo-2 (**c**) hydrophones (N_FFT_ is 2048 and 4096 samples, respectively, Hamming window, 50% overlap); (**d**,**e**) Instance of a single speedboat recorded with the SNAP (**d**) and Nemo-1 hydrophone (**e**); (**f**) Spectral probability density plot (0.03–8 kHz) and corresponding root-mean-square (RMS) levels of the PSD for the entire SNAP recording shown in (**a**); (**g**) As in (**f**), but produced from the Nemo-1 data.

**Figure 5:**
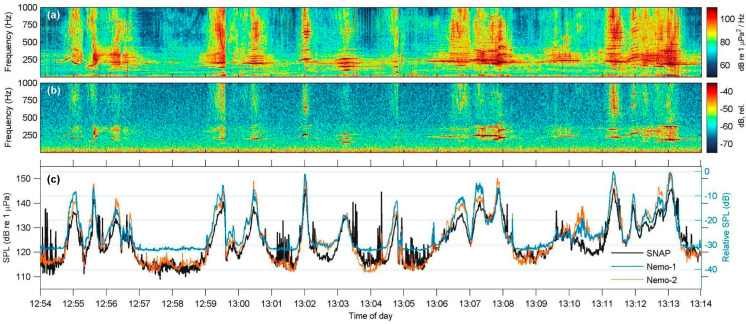
Speedboat traffic for the same 20 min-long segment presented at Figure 3, as recorded with the SNAP and the Nemo-1 hydrophones; (**a**) 20 min-long power spectral density (PSD) spectrogram produced from the SNAP data, zoomed at the 0.03–1 kHz band (N_FFT_ = 4096 samples, Hamming window, 50% overlap); (**b**) As in (**a**), but recorded with the Nemo-1 hydrophone (N_FFT_ = 2048 samples, Hamming, 50% overlap), (**c**) Corresponding root-mean-square (RMS) received sound pressure levels (SPL, 0.03–8 kHz); RMS window length is 1 s for all devices.

A total of 182 speedboat instances were identified during the 3 h recording period at the Marathonisi site, corresponding to a heavy traffic density of about 60 speedboat passages per hour. Figure 4 shows a typical 20 min segment of this session, concurrently recorded with the SNAP, Nemo-1, and Nemo-2 hydrophones. The synchronized spectrogram plots show that the low-cost hydrophones can adequately record speedboat vessel traffic in shallow waters, both in terms of boat counts and overall received sound pressure levels (Figure 5). When compared to the 182 instances identified in the SNAP dataset, only 8 passages could not be visually detected on the Nemo data, which corresponded to distant (>300 m) and/or slow-moving, low-frequency inboard vessels. This introduced an underestimation of less than 4.5% of the heavy leisure traffic of the day. As illustrated in the spectrogram plots and in the PSD of the recordings (Figure 4f,g), the low-cost hydrophones have poor reception abilities at frequencies below 200 Hz, are more effective in the mid-to-high frequency range (>1 kHz), and have resonance peaks around 3 kHz that saturate their spectrograms.

Received sound pressure level values at all three hydrophones were computed (Figure 5) for the same 20 min segment presented in Figure 4. Apart from a few SPL peaks where the SNAP hydrophone picked up short-duration broadband crackling sounds that were not registered by the Nemo devices (e.g., at 12:54:00, 13:03:30, 13:04:20, 13:09:40), the uncalibrated received pressure levels at both Nemo hydrophones were in good agreement with the reference SNAP. Overall, the comparative results show that even though the low-cost hydrophones have limited response at lower frequency bands, they are able to record events even at 250 Hz (Figure 5b), provided the source is a high-energy sound such as a close-range passing speedboat.

**Figure 6:**
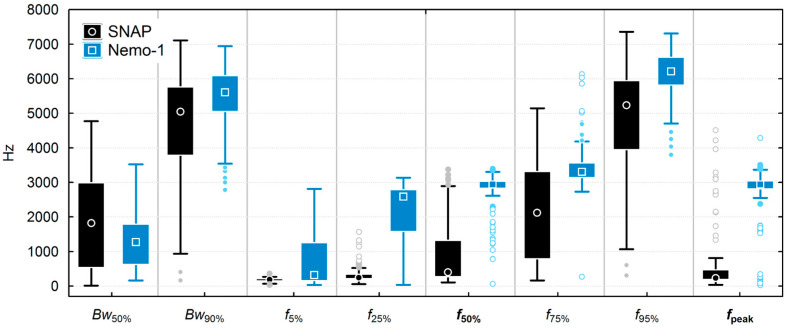
Boxplot of energy-weighted bandwidth and frequency descriptors, calculated from all 182 speedboat instances (30–8000 Hz) at Marathonisi for the SNAP and Nemo-1 hydrophones. Boxes and whiskers denote the interquartile and non-outlier range, respectively, while the center marker denotes the median.

Quantitative spectral descriptors (30–8000 Hz) were also calculated for the 182 speedboat instances recorded at Marathonisi (NMPZ) using SNAP and Nemo-1 hydrophones, and are displayed in Figure 6 as box plots. The Nemo-1-derived descriptors are biased towards higher frequencies, both due to the resonant frequency at 3 kHz that makes peak (*f*_peak_) and 50% frequency (*f*_50%_) metrics centered at this hotspot, and the low sensitivity at low-frequency sounds that do not contribute energy to the Nemo-1 selections. As a result, the energy-weighted bandwidth metrics have a smaller range than those of the SNAP, while all other frequency metrics of speedboats are overestimated.

Summary descriptive statistics of selected temporal and frequency metrics are reported in Table 2 for the SNAP and Nemo-1 hydrophones. All descriptors were statistically different between the SNAP and Nemo-1 hydrophones (Mann–Whitney U-test, *p* < 0.01), except for the energy-weighted duration (*dt*_90%_), the mean of which differed between devices by only 2.1 s.

### 3.3. Recordings of Shipping Noise at Agrilia Site

**Figure 7:**
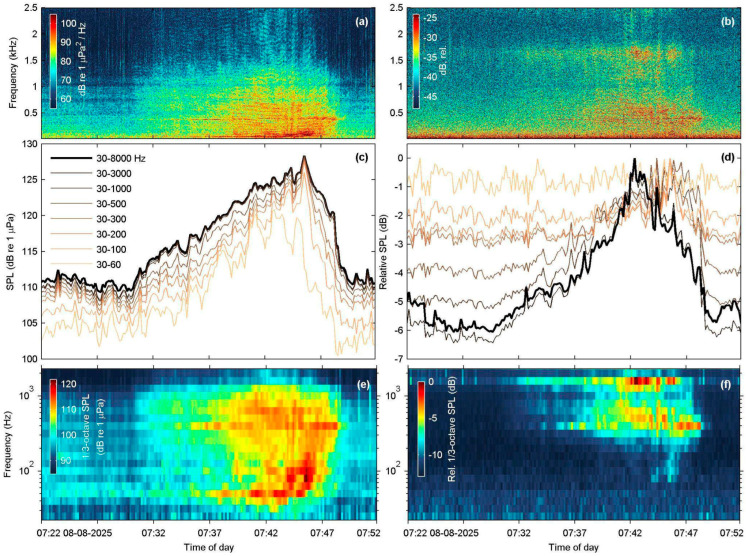
Concurrent recording of a passenger ferry boat (length: 195 m) at the Agrilia site, obtained with the SNAP and Nemo-1 hydrophones; the ferry was northbound to Mytilene port, and its distance to the acoustic station was approximately 2 km at the nearest point. (**a**) Power spectral density (PSD) spectrogram of the SNAP recording (N_FFT_ = 16,384 samples, Hamming window, 50% overlap); (**b**) As in (**a**), but for the Nemo-1 hydrophone (N_FFT_ = 10,240, Hamming window, 50% overlap); (**c**) Root-mean-square (RMS) received sound pressure levels (SPL) by the SNAP hydrophone, averaged over 10 s segments across different frequency bands; (**d**) As in (**c**), but for the Nemo-1 hydrophone; (**e**) Underwater noise measurements with the SNAP hydrophone across 1/3-octave frequency bands (0.03–2.5 kHz, RMS), computed over 10-s segments; (**f**) As in (**e**), but for the Nemo-1 hydrophone.

The one-hour recording session at Agrilia captured the passage of a northbound ferry heading to Mytilene port, at an approximate distance of 2 km from the acoustic station. Excluding a small (5 m) outboard boat that was conducting regular maintenance work at the nearby aquaculture facility, no other vessel was visible in the vicinity. The concurrent recordings and corresponding noise measurements of this event are displayed in Figure 7, both for the reference, calibrated SNAP and the low-cost, uncalibrated Nemo-1 hydrophone.

The PSD spectrogram plots (Figure 7a,b) show that the event is clearly registered by the SNAP device across the full spectrum examined. The event is also discernible at the Nemo-1 spectrogram and appears overall similar to that of SNAP, although displayed more faintly (Figure 7b). The received sound pressure levels recorded by the SNAP and Nemo-1 indicate a difference in the frequency response of the two hydrophones. The reference SNAP device employs the HTI-96-Min hydrophone, which has a flat frequency response from 2 Hz to 30 kHz, as per the manufacturer. It therefore registers a monotonic increase in received SPL values for each progressively broader frequency band, and, when compared to the approximately 112 dB re 1 μPa baseline prior to the ferry passage (Figure 7c, 07:22–07:30), it registers a maximum 17 dB re 1 μPa increase in SPL at the closest point of the ferry. On the other hand, Nemo-1 is effectively unresponsive at low frequencies below 200 Hz, where most of the ferry’s radiated noise is, registering an overall SPL increase of only 6 dB (relative) throughout the event (Figure 7d). This is also reflected in the RMS noise measurements at 1/3 octave frequency bands (Figure 7e,f), where the Nemo-1 1/3-octave bands begin to contain acoustic energy above approximately 150 to 200 Hz.

### 3.4. Recordings at Mytilene Port

**Figure 8:**
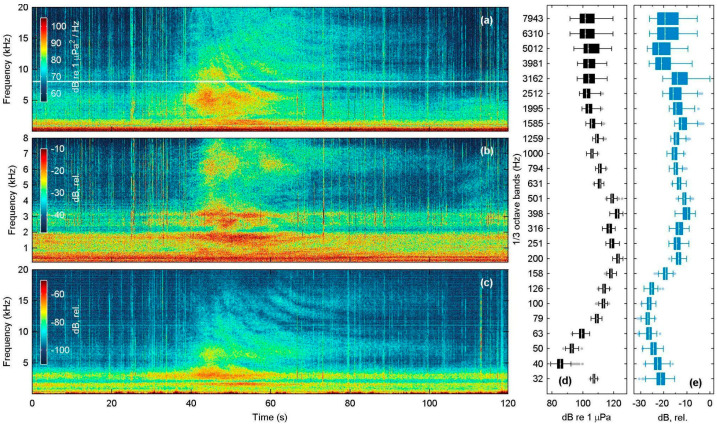
PSD spectrograms of a single inboard passenger vessel departure from the port of Mytilene, eastbound towards the port of Ayvalik (Turkey), accompanied by RMS measurements of ambient noise levels across 1/3-octave bands from 32 Hz up to 8 kHz; the white line in panel (**a**) denotes the 8 kHz upper frequency limit of the Nemo-1 handheld recorder. (**a**) Spectral density (PSD) spectrogram produced from the SNAP data (N_FFT_ = 8192 samples, Hamming window, 50% overlap); (**b**,**c**) As in (**a**), but recorded with the Nemo-1 and Nemo-2 hydrophones (N_FFT_ is 2048 and 8192 samples, respectively, Hamming, 50% overlap). (**d**,**e**) Boxplots of 1/3-octave RMS noise measurements for the SNAP (**d**) and Nemo-1 (**e**) hydrophones; lines denote the median, boxes and whiskers show the interquartile and non-outlier range, respectively.

For the recordings conducted at the port of Mytilene, PSD spectrograms of a single inboard vessel departure (40 m in length) were computed for the SNAP, Nemo-1, and Nemo-2 hydrophones, accompanied by RMS noise measurements for all 1/3-octave frequency bands from 32 Hz to 8 kHz (Figure 8). The results show similar patterns to those observed at Agrilia and Marathonisi, i.e., the Nemo low-cost hydrophones are able to amply register the high energy event at mid to high frequencies, but perform poorly below 200 Hz and systematically peak at the resonant frequency of about 3 kHz (Figure 8e, 1/3-octave band centered at 3162 Hz).

### 3.5. Long-Term Recordings of the Coastal Soundscape at Villa Site

**Figure 9:**
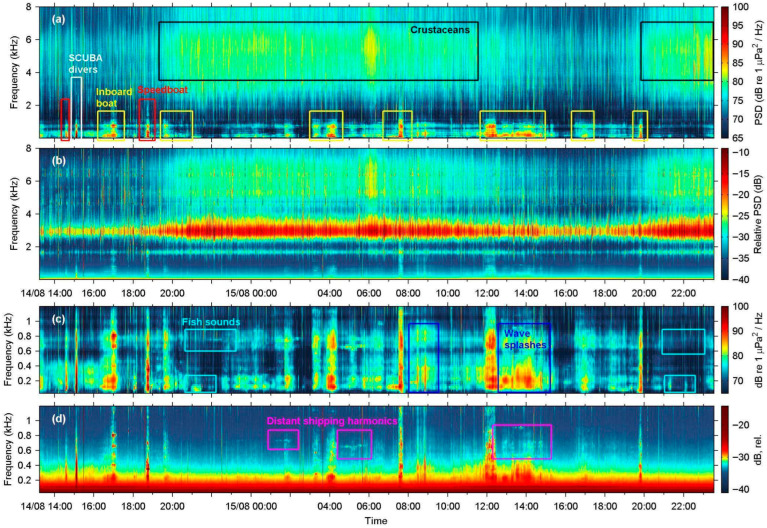
Long-term spectral average plots (LTSA, 30 s averaging window) for the 34.3 h of continuous recordings at Villa site, marked with color-coded annotations of main contributors to the soundscape; (**a**) LTSA (N_FFT_ = 4096 samples) for the reference SNAP, cropped to a maximum frequency of 8 kHz; (**b**) As in (**a**), but for Nemo-1 hydrophone (N_FFT_ = 1024); (**c**,**d**) As in (**a**,**b**), but cropped to the low-frequency spectrum ≤ 1 kHz; time is labeled as day/month hour: minute.

**Figure 10:**
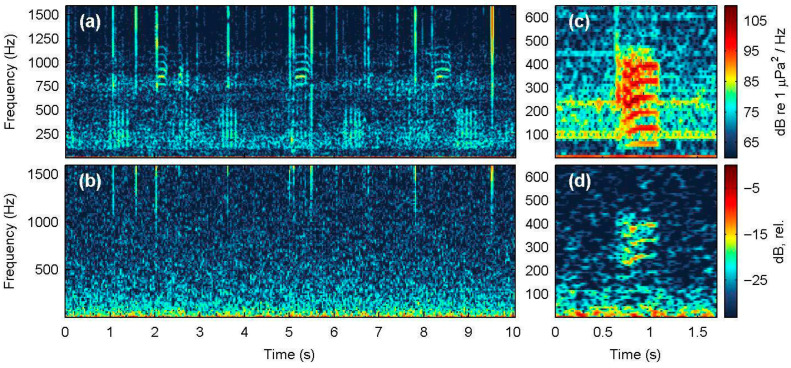
(**a**) Spectrogram of fish sounds recorded with the SNAP hydrophone (14 August 2025, 20:49 local time), showing the ‘kwa’ sounds (600–1200 Hz) and pulse trains (<500 Hz) associated with *Scorpaena* spp. and sciaenids (e.g., *Sciaena umbra*), respectively (N_FFT_ = 8192 samples, Hamming window, 90% overlap). (**b**) Concurrent recording with the Nemo-1 hydrophone of the same time segment shown in (**a**) (N_FFT_ = 1024, Hamming, 50%); note the absence of all fish sounds recorded with the SNAP. (**c**,**d**) Fish-derived growling sound produced in close proximity to the acoustic station, as recorded with the SNAP (**c**) and the Nemo-1 (**d**) hydrophones (14 August 2025, 18:05 local time); the sound source is likely a dusky grouper [48].

Long-term spectral average plots were produced to summarize the continuous recordings (34.3 h) at the Villa site obtained with the SNAP and Nemo-1 hydrophones (Figure 9). The diurnal cycle of crustacean sound activity at high frequencies is prominent in both plots, as are the various small-scale fishing inboard vessels that frequent these coastal waters. The Nemo-1 output is highest at the resonant frequency of 3 kHz, effectively saturating the spectrogram during the night, when crustacean snapping sounds mostly contribute to the soundscape. At lower frequencies around 300 Hz to 1 kHz (Figure 9c,d), most high-energy instances were recorded by Nemo-1, albeit signals below 200 Hz are practically absent from its spectrogram. Although the entire acoustic dataset was scrutinized for fish sounds that were regularly present in the SNAP data, especially during dusk, only three such low-frequency instances were spotted in the Nemo-1 series, corresponding to sounds produced by fish that were apparently very close to the acoustic station (Figure 10).

## 4. Discussion

Passive acoustics is a key, non-invasive tool for measuring, monitoring, and identifying the sources of sound in underwater environments [2,3,51,53]. It enables scientists to study the acoustic behavior and ecology of marine animals [54,55,56,57], the levels of anthropogenic noise [10,20,58] and its impacts on marine biota [14,15,17,59,60], and the geophysical or atmospheric processes that introduce acoustic energy underwater [61,62,63,64,65]. Collectively, these sounds contribute to the acoustic complexity of marine environments [66,67] and constitute the underwater ‘soundscape’ [68], formally defined as the “characterization of the ambient sound in terms of its spatial, temporal and frequency attributes, and the types of sources contributing to the sound field” [69]. By utilizing PAM tools over large spatiotemporal scales or in short-term studies at specific sites, researchers can monitor marine animals and their habitats and provide evidence-based assessments to policymakers and MPA managers [19,27,70,71]. Some PAM applications are particularly demanding in equipment specifications, data storage needs, calibration accuracy, deployment duration, and data collection protocols. Such applications include long-term underwater noise monitoring [20,58], deep water high frequency autonomous hydrophones [72], in situ validation of shipping noise models [73], or source level measurements of animals [35,74] and anthropogenic sound sources [75,76]. However, many other passive acoustic studies can be implemented without high-end equipment, e.g., acoustic detection of animals in the wild [39,77,78,79,80] or in controlled environments [38,81,82], portable audio–video underwater platforms [83], speedboat traffic assessment at coastal sites or MPAs [22,84], monitoring aquaculture sites [42], or bioacoustics education and citizen science projects [85].

In most cases, however, pressure resistance and waterproofing requirements render underwater scientific equipment more expensive than its terrestrial counterparts [37], and hydrophones are not an exception to this trend. There are various hydrophone types [86] and autonomous acoustic recorders [87] depending on study needs, but even the low-end devices cost from several hundred to a few thousand euros. Moreover, the hydrophone sensor is not the only apparatus required for passive acoustic data acquisition, as the recording device adds further costs that can, in total, hinder initiatives with limited budgets. The possibility of losing or damaging such expensive equipment in the field can also restrict operations, while the logistics and costs of deploying and maintaining multiple acoustic stations across large spatial scales are always a consideration in PAM applications. Thus, low-budget and/or large-scale initiatives particularly benefit from inexpensive passive acoustic devices that can support broad monitoring of underwater soundscapes. This potential is especially relevant for citizen science projects, where resources should be expanded not only to cover broad areas but also to engage large numbers of participants.

To this end, we have developed and experimentally assessed a low-cost passive acoustic toolkit within the scope of the NEMO-Tools project, aiming to freely distribute custom-made hydrophones coupled with low-budget recorders as a complete acoustic sampling toolkit for citizen scientists, such as fishers, boaters, scouts, naturalists, and students. This will enable the collection of large amounts of passive acoustic data through the active involvement of citizens and the launch of multiple monitoring initiatives, while also promoting acoustic education and supporting research in schools and science projects with limited resources. Our proposed toolkit has a total cost of approximately 50 € (including the handheld recorder), and, taking into account its limitations, it can serve as an effective alternative to high-end commercially available equipment.

The in situ field tests at four sites demonstrated that Nemo hydrophones showed mechanical endurance, produced consistent-quality recordings, and registered most sounds detected by their scientific counterpart, apart from some low-intensity or low-frequency events. Specifically, during the Marathonisi deployment at NPMZ, both Nemo hydrophones successfully characterized vessel traffic inside an MPA frequented by recreational speedboats, in high agreement with the SNAP reference hydrophone. Notwithstanding the resonant peaks at 3 kHz and poor sensitivity below 200 Hz, the spectral structure and energy distribution of speedboat events were preserved (Figure 4d,e) at frequencies up to 24 kHz (Figure 4c). At the other test sites, anthropogenic sound sources were also adequately identified (Figure 5, Figure 8 and Figure 9), with the exception of lower frequency bands from distant sources (Figure 7f). The long-term deployment at Villa also showed that the low-cost Nemo hydrophones can be used for continuous recordings over multiple days and were able to identify most contributors to the coastal soundscape, even in the time-averaged LTSA plots (Figure 9).

However, all field evaluations highlighted clear performance limitations of the Nemo hydrophones for low-frequency events. Low-intensity fish sounds that are typically below 1 kHz were not recorded by Nemo-1 at Villa (Figure 10a,b), while the eight missed vessel passages at Marathonisi (NPMZ) were from distant (>300 m) inboard vessels. Moreover, all shipping noise TOLs below 200 Hz were underestimated because of the poor sensitivity of the Nemo PZ sensor at low frequencies, both in the close-range recording at Mytilene port (Figure 8e) and in the distant ferry at Agrilia (Figure 7f). Another downside of the PZ sensor used was its strong resonant frequency at 3 kHz, evident in all recordings. This saturated the Nemo spectrograms and LTSAs around 2.6 to 3.1 kHz and introduced systematic bias in most spectral descriptors and noise TOLs. Lastly, the single disk-shaped acoustic sensor used renders the Nemo hydrophones quite directional. Early ad hoc tests during construction showed that they performed better when directed towards the sound source. In our current design, the PZ housing trapped a small air pocket, which, in combination with the brass pipe fitting, caused the unit to align nearly parallel with the sea surface when suspended by its audio cable. This orientation is actually advantageous for many use cases, since it points to the water column rather than the seafloor, but refining the design for consistent orientation in drop-off deployments would improve reliability.

When compared to other similar low-cost hydrophone designs [38,39,41], Nemo hydrophones share the advantages of affordable, custom designs. However, commercial hydrophones are generally designed to have their resonant frequencies way above their intended operational range [86]. Piezoelectric disks such as those used in our low-cost design and in other similar works [38,39,40,41] do not adhere to this principle and present resonant frequencies at ranges typically below 5 kHz, depending on the PZ disk type and hydrophone housing. Past studies do not report recording analysis below 2 kHz [38], and our findings show that, while present, resonance artifacts do not negate the extraction of meaningful information at low to mid frequencies.

Future upgrades of the Nemo hydrophones should focus on mitigating these issues and enhancing operational capabilities. We plan to experiment with multiple piezoelectric elements of different diameters in the sensor design to improve low-frequency response and reduce blind spots attributed to directionality, starting by employing larger-in-diameter PZ disks, as past experiments seem to indicate better reception capabilities for the applications discussed herein [40]. Our current design was kept simple (relying on the recorder’s built-in gain options), but incorporating a dedicated, inexpensive preamplifier circuit could significantly boost the signal-to-noise ratio [38]. A compensating filter could also help mitigate mid-frequency resonance peaks. Additionally, a small low-cost array could enable rudimentary localization of sound sources (through time-of-arrival differences and a multi-channel recorder) or improve detection of specific events through cross-correlation of signals. Cetacean recordings will also be conducted, given the good performance of the Nemo units at high frequencies up to 24 kHz, while calibration of an improved unit should also be pursued [39].

Overall, the Nemo hydrophones tested herein represent a viable alternative for large-scale monitoring efforts, though they cannot match the bandwidth, sensitivity, and dynamic range of professional scientific hydrophones. Despite limitations, the advantage of an easily replaceable, inexpensive device is that researchers can afford to deploy many units simultaneously, covering multiple sites or larger areas. This trade-off between quality and quantity could actually be an advantage in certain scenarios; for example, large-scale, lower-resolution datasets could be highly effective for detecting broad spatial and temporal patterns of underwater noise that might otherwise go unnoticed in smaller, high-precision studies; while even if lower in resolution, could allow researchers to compare seasonal trends in human uses across different areas and regions. In conclusion, our study demonstrates that a carefully designed, inexpensive hydrophone toolkit can greatly augment passive acoustic monitoring. By empowering citizen scientists and resource-limited programs, a far greater volume of data on underwater soundscapes can be collected, ultimately supporting better-informed management of noise in marine-protected areas and beyond.

## Figures and Tables

**Table 1:** Acoustic station information and recording settings per test site; all devices recorded continuously and saved the acoustic data in 16-bit uncompressed audio (WAV) format (*R*_t_: recording duration; *f*_s_: sampling frequency). Dates are shown as dd-mm-yyyy.

Site	Coordinates	Date	Depth (m) ^1^	*R*_t_ (min)	Device	Recorder	*f*_s_ (kHz)
Marathonisi	37.68703° N 20.86630° E	26-06-2025	3 (3)	180	Nemo-1	Philips	16
					Nemo-2	Tascam	48
					SNAP	(embedded)	48
Agrilia	39.00706° N 26.60246° E	08-08-2025	7 (35)	60	Nemo-1	Philips	16
					Nemo-2	M-Audio	48
					SNAP	(embedded)	96
Mytilene port	39.10287° N 26.55898° E	10-06-2025	4 (4)	30	Nemo-1	Philips	16
					Nemo-2	Tascam	44.1
					SNAP	(embedded)	48
Villa	39.01329° N 26.55154° E	14/15-08-2025	6 (6)	2060	Nemo-1	Philips	16
					Nemo-2	M-Audio	44.1
					SNAP	(embedded)	96

^1^ Numbers in parentheses denote seafloor depth below the acoustic station.

**Table 2:** Summary of duration and frequency measurements for the 182 speedboat instances (30–8000 Hz) recorded at Marathonisi (NMPZ); frequency descriptors are rounded to the nearest integer for clarity (SD: standard deviation, IQR: interquartile range).

Descriptor	Statistic	SNAP	Nemo-1
90% Duration	Mean ± SD	23.3 ± 14.1	21.2 ± 14.3
*dt*_90%_ (s)	Range	4.6–113.3	3.5–111.3
	IQR	15.4–27.0	12.9–25.3
90% Bandwidth	Mean ± SD	4467 ± 1455	5484 ± 822
*Bw*_90%_ (Hz)	Range	164–7113	2781–6945
	IQR	3785–5766	5055–6086
95% Frequency	Mean ± SD	4838 ± 1476	6155 ± 626.2
*f*_95%_ (Hz)	Range	305–7359	3797–7312
	IQR	3949–5941	5812–6617
Peak frequency	Mean ± SD	502 ± 765	2626 ± 894
*f*_peak_ (Hz)	Range	35–4512	31–4289
	IQR	187–469	2812–3039
Center frequency	Mean ± SD	856 ± 803	2802 ± 503
*f*_50%_ (Hz)	Range	105–3375	62–3398
	IQR	269–1324	2836–3031
5% frequency	Mean ± SD	170 ± 59	671 ± 632
*f*_5%_ (Hz)	Range	35–363	31–2812
	IQR	152–211	156–1258

## Data Availability

The original contributions presented in this study are included in the article. Further inquiries can be directed to the corresponding author.

## Abbreviations

The following abbreviations are used in this manuscript:

DIY	Do-it-yourself
FFT	Fast Fourier transform
IQR	Interquartile range
LTSA	Long term spectral average
MPA	Marine-protected area
MSFD	Marine Strategy Framework Directive
NMPZ	National Marine Park of Zakynthos
PAM	Passive acoustic monitoring
PSD	Power spectral density
PVC	Polyvinyl chloride
PZ	Piezoelectric
RMS	Root-mean-square
SD	Standard deviation
SPL	Sound pressure level

## Appendix A

**Table A1:** List of components used for the construction of the Nemo hydrophones; reusable components across multiple units are marked with an asterisk (*).

Component	Function	Price (€)
Piezoelectric disk (Bestar: FT-35T-2.6A1, 35 mm) - Resonant Frequency: 2.6 0.5K Hz - Resonant Impedance: 200 max ohm - Capacitance: 33 30%nF - Input Voltage: Max. 30 Vp-p - Metal Material: Brass	Acts as the acoustic element of the device	1.5/unit
Audio cable (10 m)	Connects the PZ disk to the recording device	5/unit
⌀60 mm plexiglass disk (4 mm)	Is attached to the PZ disk and acts as a lid to the hydrophone’s enclosure	3/unit
PVC threaded nipple	Engulfs the acoustic element (housing)	0.85/unit
Brass pipe fitting	Threaded on the PVC extension, it closes the recorder-side of the hydrophone and helps the audio cable exit the watertight enclosure. Additionally, its weight keeps the hydrophone submerged underwater	6/unit
Heat shrink tubing *	Used for sealing and abrasion resistance at the point where the audio cable exits the enclosure	0.15/m
Sikaflex-291i *	Used for sealing the front and back side of the hydrophone	12/300 mL
Liquid teflon *	Used for sealing the connection between the brass fitting and the PVC thread	11/100 mL
Teflon tape *	Used for sealing the connection between the brass fitting and the PVC thread	0.5/10 m
Cyanoacrylate glue *	Used for attaching the PZ disk on the plexiglass disk, and for attaching the plexiglass disk on the PVC extension	2.5/unit
